# *PLA2G16* is a mutant p53/KLF5 transcriptional target and promotes glycolysis of pancreatic cancer

**DOI:** 10.1111/jcmm.15832

**Published:** 2020-09-27

**Authors:** Wei Xia, Hansong Bai, Ying Deng, Yi Yang

**Affiliations:** ^1^ Department of Endocrinology Sichuan Provincial People's Hospital University of Electronic Science and Technology of China Chengdu China; ^2^ Cancer Center Sichuan Provincial People's Hospital University of Electronic Science and Technology of China Chengdu China

**Keywords:** glycolysis, KLF5, mutant p53, pancreatic cancer, PLA2G16

## Abstract

PLA2G16 is a member of the phospholipase family that catalyses the generation of lysophosphatidic acids (LPAs) and free fatty acids (FFAs) from phosphatidic acid. In the current study, we explored the functional role of PLA2G16 in pancreatic adenocarcinoma (PAAD) and the genetic/epigenetic alterations leading to its dysregulation. Bioinformatic analysis was performed using data from The Cancer Genome Atlas (TCGA), Genotype‐Tissue Expression (GTEx) and the Human Protein Atlas (HPA). Then, PANC‐1 and MIA‐PaCa‐2 cells harbouring *TP53* mutations were used for cellular and animal studies. Results showed that *PL2G16* expression was significantly up‐regulated in PAAD tissue and was associated with unfavourable survival. *PLA2G16* inhibition suppressed pancreatic cell growth in vitro and in vivo and also inhibited aerobic glycolysis. Bioinformatic analysis indicated that *KLF5* was positively correlated with *PLA2G16* expression in PAAD tumours with *TP53* mutation. *TP53* or *KLF5* inhibition significantly reduced *PLA2G16* expression at both mRNA and protein levels. Dual‐luciferase and chromatin Immunoprecipitation‐quantitative polymerase chain reaction assays showed that KLF5 directly bound to the PLA2G16 promoter and activated its transcription. Co‐immunoprecipitation assay indicated that mutant p53 had a physical interaction with KLF5. Inhibition of mutant p53 impaired the transcriptional activating effects of KLF5. In PAAD cases in TCGA, *PLA2G16* expression was positively correlated with its copy number (Pearson's *r* = 0.51, *P* < 0.001), but was strongly and negatively correlated with the methylation level of cg09518969 (Pearson's *r* = −0.64, *P* < 0.001), a 5’‐cytosine‐phosphodiester bond‐guanine‐3’ site within its gene locus. In conclusion, this study revealed a novel mutant p53/KLF5‐PLA2G16 regulatory axis on tumour growth and glycolysis in PAAD.

## 1 INTRODUCTION

Pancreatic adenocarcinoma (PAAD) is among the most aggressive and lethal malignancies with very poor prognosis.^1^ Although with significant advances in therapeutic strategies, the 5‐year overall survival (OS) remains around 6%.^1^ Reprogrammed energy metabolism has been characterized as an important factor leading to aggressive invasion and early metastasis of PAAD.^2^, ^3^ Therefore, uncovering the molecular mechanisms associated with reprogrammed energy metabolism will provide novel insights into the development of targeted therapy.

PLA2G16 is a member of the phospholipase family and is also known as phospholipase A and acyltransferase 3 and Ha‐RAS like suppressor 3. It was initially identified as a potential suppressor due to its suppressive effects on Ras‐mediated transformation and proliferation in cultured cells.^4^, ^5^ However, as a phospholipase, it catalyses the generation of lysophosphatidic acids (LPAs) and free fatty acids (FFAs) from phosphatidic acid.^6^ LPA is a bioactive lipid that activates multiple tumour‐promoting signalling pathways, such as ERK,^7^ YAP,^8^ RAC,^9^ MAPK^10^ and PLC/DGK^11^ pathways. Besides, FFAs such as arachidonic acid can be converted into prostaglandins by cyclooxygenases, thereby participating in cancer progression.^12^, ^13^, ^14^, ^15^ Therefore, PLA2G16 may play a critical role in tumour progression by regulating metabolic pathways.

One previous study found that PLA2G16 mediates the gain‐of‐function activities of mutant p53 in osteosarcoma.^16^ Mutant p53 binds to E26 transformation‐specific motifs in the PLA2G16 promoter indirectly via ETS2, thereby promoting its expression.^16^ *TP53* mutation is quite common in multiple types of cancer, including PAAD. Mutant p53s have lost the tumour‐suppressing effect of wild‐type p53 and also gain functions that facilitate tumour progression.^17^ In the current study, we explored the functional role of PLA2G16 in PAAD, as well as genetic/epigenetic alterations leading to its dysregulation. We showed that *PLA2G16* is up‐regulated in PAAD and associated with poor prognosis. Its up‐regulation results in enhanced glycolysis and growth of PAAD cells. Moreover, we showed that *PLA2G16* is a transcriptional target of KLF5. Mutant p53 enhances the binding of KLF5 to the *PLA2G16* promoter. DNA copy number amplification and promoter hypomethylation might also contribute to *PLA2G16* up‐regulation in PAAD.

## 2 MATERIALS AND METHODS

### 2.1 Data analysis in TCGA Pan‐Cancer and GTEx‐Pancreas

The RNA‐seq, genetic mutation, clinicopathological and survival data of patients with primary PAAD in TCGA Pan‐Cancer were downloaded from the UCSC Xena browser (https://xenabrowser.net/).^18^ The screening process of the illegible patients was summarized in supplementary Figure 1. RNA‐seq data in these two datasets were pre‐normalized and represented as log2 (TPM + 0.001). Survival data, including OS, disease‐specific survival (DSS) and progression‐free survival (PFS), were extracted for survival analysis. A total of 178 PAAD cases with OS and PFS data were included. DSS data were available among 176 of them.

**FIGURE 1:**
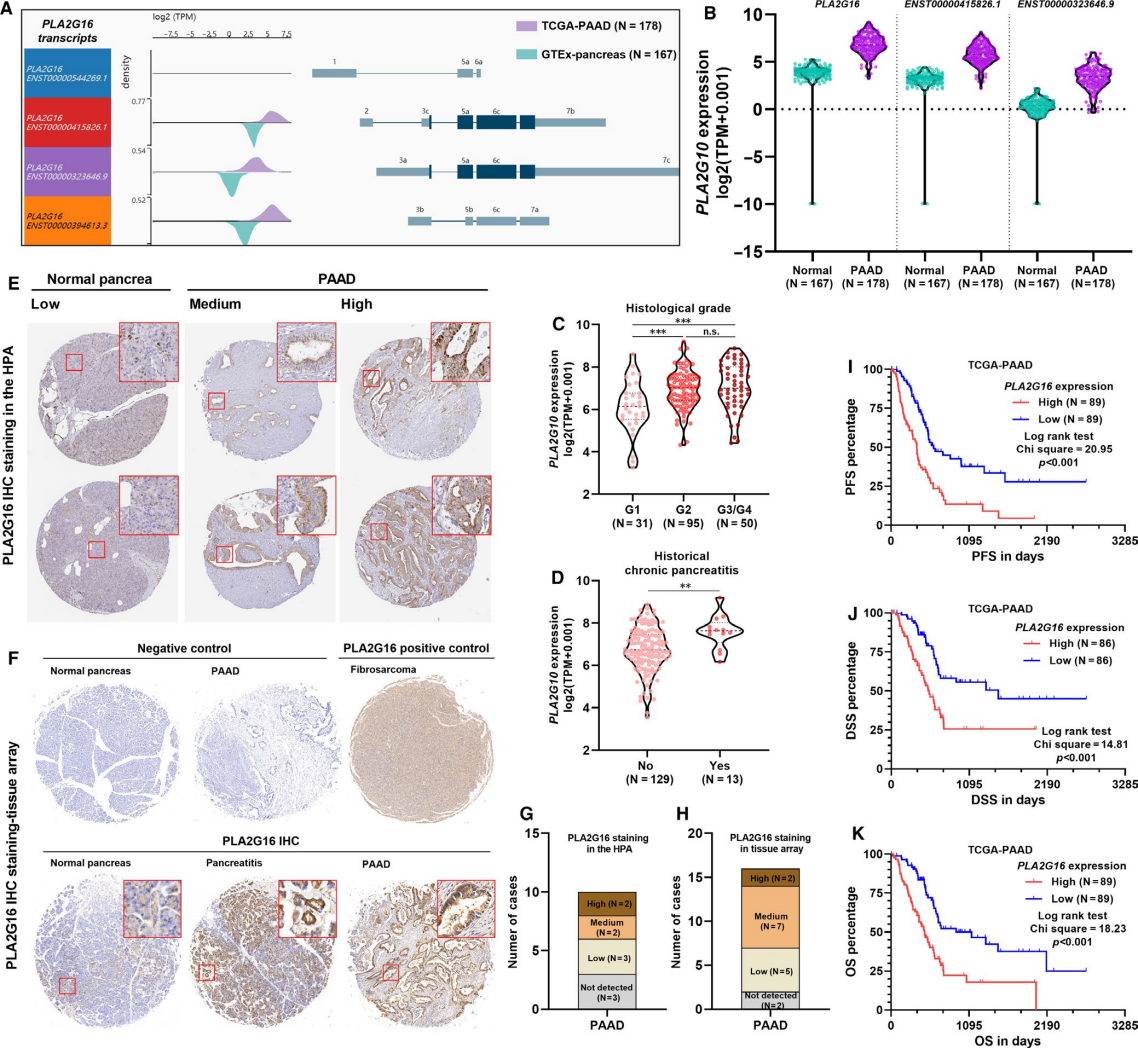
Aberrant *PLA2G16* expression was associated with unfavourable survival of pancreatic adenocarcinoma (PAAD). A and B, Schematic diagram (A) and violin chart (B) showing the exonic and intronic structure of *PLA2G16* transcripts and the expression of the protein‐coding transcripts in primary PAAD cases in The Cancer Genome Atlas (TCGA) and normal pancreas in GTEx. C and D, Comparison of *PLA2G16* mRNA expression in different grades of PAAD (C) and between the cases with or without historical chronic pancreatitis (D) in TCGA. E, Representative images of IHC staining of PLA2G16 in normal pancreas and PAAD tissues. Image credit: Human Protein Atlas, from https://www.proteinatlas.org/ENSG00000176485‐PLA2G16/tissue/pancreas and https://www.proteinatlas.org/ENSG00000176485‐PLA2G16/pathology/pancreatic+cancer#ihc. F, Representative images of IHC staining of PLA2G16 in normal pancreas, pancreatitis and PAAD in human tissue array. PBS was used as negative control. Fibrosarcoma tissue was used as positive control. G and H, Summary of PLA2G16 protein expression score of 10 PAAD cases examined in the Human Protein Atlas (G) and 16 PAAD cases in the tissue array (H). I‐K, K‐M survival analysis of progression‐free survival (PFS) (I), disease‐specific survival (DSS) (J) and overall survival (OS) (K) in PAAD cases extracted from TCGA Pan‐Cancer, by median *PLA2G16* expression separation

### 2.2 Immunohistochemical data of PLA2G16 expression

PLA2G16 immunohistochemical (IHC) staining in normal pancreas and in PAAD cases was examined using data provided by the Human Protein Atlas (HPA; https://www.proteinatlas.org/).^19^, ^20^ Three normal and 10 PAAD cases were examined. The primary antibody used was HPA058997 (Sigma‐Aldrich, St. Louis, MO, USA). Protein expression was manually scored in the database, based on a combined assessment of staining intensity (negative, weak, moderate or strong) and the fraction of stained cells (<25%, 25%‐75% or >75%).

Human PAAD tissue array, which included 16 primary PAAD tissue, four pancreatitis and four tumour adjacent normal pancreatic tissues and human sarcoma tissue array were purchased from Alenabio (Xian, China). IHC staining was conducted as described previously.^21^ Anti‐PLA2G16 was used (1:200, **#**MA5‐26290; Thermo Fisher Scientific, Waltham, MA, USA), with PBS served as the negative control. PLA2G16 staining in fibrosarcoma was used as a positive control.

### 2.3 Single‐gene Gene set enrichment analysis

Gene set enrichment analysis (GSEA) was performed between PAAD cases from TCGA with high (top 50%) and low (bottom 50%) *PLA2G16* expression. GSEA parameters setting followed the recommendation from the Broad Institute Gene Set Enrichment Analysis website.^22^ The Molecular Signatures Database v7.0 was used for running GSEA, within the Hallmark gene sets (H).^23^ The number of permutations was set to 1000. NOM *P* < 0.05 and adjusted *q*‐values (FDR) < 0.25 were considered significant.

### 2.4 Cell culture and treatment

Human PAAD cell lines (PANC‐1 and MIA‐PaCa‐2) harbouring *TP53* mutations were purchased from the American Type Culture Collection (ATCC, Manassas, VA, USA). PANC‐1 cells were cultured in Dulbecco's Modified Eagle's Medium (DMEM), containing foetal bovine serum (FBS) at a final concentration of 10%, while MIA‐PaCa‐2 cells were cultured in DMEM, with 10% FBS and 2.5% horse serum, at 37°C in 5%CO_2_. Lentiviruses carrying *PLA2G16* shRNA (shRNA#1:5ʹ‐TCTATGTTGGCGATGGATATG‐3ʹ and shRNA#2:5ʹ‐GACAAGTACCAGGTCAACAAC‐3ʹ), *TP53* shRNA (shRNA#1:5ʹ‐TCAGACCTATGGAAACTACTT‐3ʹ and shRNA#2:5ʹ‐CGGCGCACAGAGGAAGAGAAT‐3ʹ) and *KLF5* shRNA (shRNA#1:5ʹ‐CCTATAATTCCAGAGCATAAA‐3ʹ and shRNA#2:5ʹ‐GCTGTAATGTATATGGCTTTA‐3ʹ) were constructed by HanBio Technology (Shanghai, China). Lentiviral vector for *KLF5* (NM_001730.5) overexpression (lenti‐KLF5) was also generated. Lentiviral plasmids with scramble shRNA (shNC) and empty vector were used as controls. Shuttle plasmids for gene knockdown and overexpression were pHBLV‐CMVIE‐IRES‐ZSGreen and pHBLV‐U6‐ZSGreen plasmids, respectively.

Lentiviral particles were amplified by co‐transfecting with the lentivirus packaging plasmids (pSPAX2 and pMD2G; HanBio Technology) in 293T cells according to the manufacturer's instructions. 48 hours after transfection, supernatants were harvested, centrifuged at 300 *g* for 10 minutes at 4°C and filtered through a 0.45 μm filter. Finally, the supernatant was ultracentrifuged at 120 000× g for 2 hours at 4°C, dissolved in PBS after the removal of the supernatant, and stored at −80°C. Cells were infected with lentiviruses at a multiplicity of infection of 10, with the presence of 8 μg/mL polybrene.

### 2.5 Western blotting

Cells were harvested, washed with ice‐cold PBS and lysed in RIPA buffer supplemented with protease and phosphatase inhibitors for 10 minutes. Protein concentrations were determined by using a BCA Protein Assay Kit (Beyotime, Wuhan, China). 25 μg total protein was subjected to electrophoresis in denaturing 10% SDS‐PAGE, and then transferred to polyvinylidene fluoride membranes (Millipore, Billerica, MA, USA). The membranes were then blocked with 5% non‐fat milk powder dissolved in Tris‐buffered saline with Tween‐20 for 1 hour and incubated with primary antibodies overnight at 4°C. The primary antibodies used include anti‐PLA2G16 (1:4000, **#**MA5‐26290; Thermo Fisher Scientific), anti‐p53 (1:250, #MA5‐14516; Thermo Fisher Scientific), anti‐KLF5 (1:1000, 21017‐1‐AP; Thermo Fisher Scientific). Then, the membranes were incubated with HRP‐conjugated secondary antibodies for 1 hour at room temperature. The blots were then visualized with BeyoECL Star reagent (Beyotime) and an ImageQuant LAS‐4000 imaging system (GE Healthcare, Piscataway, NJ, USA).

### 2.6 Measurement of extracellular acidification rates and oxygen consumption rate

Extracellular acidification rates (ECAR) and oxygen consumption rate (OCR) were measured using a Seahorse XFe96 (Agilent Technologies, Santa Clara, CA, USA) with the Seahorse XF Glycolysis Stress Test Kit (cat no. 103020‐100, Agilent Technologies) and Cell Mito Stress Test Kit (cat no. 103015‐100, Agilent Technologies), according to the manufacturer's instructions. In brief, 5 × 10^4^ cells were seeded into XF96 culture plates in quintuplicate 1 day before analysis. ECAR was measured under basal conditions and after sequential treatment of the PAAD cells with serial injections of glucose (10 mmol/L), oligomycin (1 μmol/L) and 2‐deoxy‐D‐glucose (2‐DG) (100 mmol/L). For OCR measurement, 1 μmol/L oligomycin, 1 μmol/L carbonyl cyanide 4‐(trifluoromethoxy) phenylhydrazone and 1 μmol/L rotenone were automatically injected into XF96 Cell Culture Microplates. ECAR and OCR were normalized to the cell number as determined by CellTiter‐Glo analysis at the end of the experiments.

### 2.7 Lactate production and glucose uptake assay

PANC‐1 and MIA‐PaCa‐2 cells were plated in a 24‐well plate at the density of 2 × 10^5^ cells/mL. Aliquots of media from each well were assessed 24 hours later for the amount of lactate present, using a lactate oxidase‐based colorimetric assay (Beyotime) according to the manufacturer's instructions. For glucose uptake assay, PANC‐1 and MIA‐PaCa‐2 cells were cultured under normoxic conditions in DMEM (glucose‐free) for 16 hours and were then incubated with DMEM (high‐glucose) for 24 hours. Then, culture medium was removed and the intracellular glucose levels were measured by a fluorescence‐based glucose assay kit (BioVision, Exton, PA, USA) according to the manufacturer's instructions.

### 2.8 Cell proliferation assay

24 hours after infection, PANC‐1 and MIA‐PaCa‐2 cells were plated into 96‐well culture plates (3000 cells/well) for cell proliferation assays, using cell count kit (CCK‐8) assay (Dojindo, Kumamoto, Japan) according to the manufacturer's protocol. In brief, 10 μL/well CCK‐8 reagent was added at 0, 24, 48, 72, 96 and 120 hours after plating, with an additional 2.5 hours incubation at 37°C. The optical density (OD) at the 450‐nm wavelength (OD450) was measured.

### 2.9 Colony formation assay

PANC‐1 and MIA‐PaCa‐2 cells (n = 500) 24 hours after infection were seeded. After cultivating for 10 days, 4% paraformaldehyde was used to fix the cells, followed by staining with 1% crystal violet. The colonies were subsequently counted.

### 2.10 Flow cytometric analysis of cell apoptosis

Cell apoptosis 48 hours after lentiviral infection was analysed using the Annexin V Apoptosis Kit‐FITC (catalog#NBP2‐29373, Novus Biologicals, Centennial, CO, USA), according to the manufacturer's instruction. Flow cytometric analysis was conducted using a FACSAria III flow cytometer (BD Biosciences, San Jose, CA, USA).

### 2.11 In vivo tumour formation assay

Animal studies were approved by the Institutional Animal Care and Use Committee of Sichuan Academy of Medical Science & Sichuan Provincial People's Hospital, China. The athymic Balb/c nude mice aged from 4 to 6 weeks were purchased from Hunan SJA Laboratory Animal Co, Ltd (Changsha, China) and were used for the in vivo tumorigenesis experiments. 1 × 10^7^ cells in 100 μL PBS were injected subcutaneously into the right hind leg of mice. Since day 3 after the injection of tumour cells, tumour growth was evaluated once every 3 days by measuring the length and the width with electronic calipers. Tumour volume was calculated as follows: volume (mm^3^) = (*L* × *W* ^2^)/2, in which *L* indicates length diameter (mm), while *W* is width diameter (mm).

### 2.12 RNA extraction and quantitative real‐time PCR

Total RNA was extracted from tissues or cultured cell lines using TRIzol reagent (Invitrogen, San Diego, CA, USA) following the manufacturer's instructions. RNA concentration was measured with NanoDrop ND‐2000 spectrophotometers (Thermo Fisher Scientific). Then, total RNA was reversely transcribed to cDNA using oligodT primers and SuperScript II reverse transcriptase (Invitrogen). Real‐time PCR was performed using SYBR Green reaction mix (Qiagen, Hilden, Germany) and analysed with the ABI PRISM 7900HT Sequence Detection System (Applied Biosystems, Foster City, CA, USA). Gene expression was normalized to the expression of β‐actin and calculated using the 2^−∆∆CT^ method.

The following primers were used as follows: human mutant *TP53*, 5ʹ‐ACAGCTTTGAGGTGCGTGTTT‐3ʹ (forward) and 5ʹ‐CCCTTTCTTGCGGAGATTCTCT‐3ʹ (reverse); human *KLF5*, 5ʹ‐AAGGAGTAACCCCGATTTGG‐3ʹ (forward) and 5ʹ‐TGGCTTTTCACCAGTGTGAG‐3ʹ (reverse); human *PLA2G16*, 5ʹ‐CCAGGTCAACAACAAACATGATG‐3ʹ (forward) and 5ʹ‐CCCGCTGGATGATTTTGC‐3ʹ (reverse); human *ACTB*, 5ʹ‐TTGTTACAGGAAGTCCCTTGCC‐3ʹ (forward) and 5ʹ‐ATGCTATCACCTCCCCTGTGTG‐3ʹ (reverse).

### 2.13 Prediction of KLF5 binding site in the promoter region of *PLA2G16*

The promoter sequence of *PLA2G16* was acquired from the *PLA2G16* promoter clone in GeneCopoeia (ID: HPRM34796, Genome = hg38; chr11‐:63615772‐63614230; length = 1543). The sequence information was provided in Figure S1. Then, the promoter sequence was scanned using JASPAR (http://jaspar.genereg.net/) to identify potential KLF5 binding sites, by setting the relative profile score threshold to 90%.

### 2.14 Dual‐luciferase assay

The integrated or truncated *PLA2G16* promoter segments were cloned in pGL3 basic vector (Promega, Madison, WI, USA). MIA‐PaCa‐2 and PANC‐1 cells were seeded in 24‐well plates at a density of 2 × 10^5^ cells per well. 24 hours later, cells were transfected with either 1 μg of empty pGL3 basic vector or the recombinant vectors with different length of *PLA2G16* promoter fragments, using Lipofectamine 3000 (Invitrogen). 0.05 μg of pRL‐CMV vector was co‐transfected to normalize the transfection efficiency. For lentivirus‐infected groups, cells were subjected to lentivirus infection 24 hours after plating and were used for dual‐luciferase assay 24 hours later. After transfection, cells were further cultured for 24 hours. Then, cells were lysed, and the activities of firefly luciferase and renilla luciferase were quantified using a dual‐specific luciferase assay kit according to the manufacturer's instruction (#E1910, Promega), with a luminometer (Promega).

### 2.15 Chromatin Immunoprecipitation‐quantitative polymerase chain reaction

Chromatin immunoprecipitation (ChIP) was conducted with the use of the Chromatin Immunoprecipitation Kit (17‐295; Merck Millipore, Boston, MA, USA), following the manufacturer's instructions. The lysates were incubated with anti‐KLF5, anti‐TP53 or IgG. Immunoprecipitated DNA was collected using Protein A beads and was purified after phenol extraction and was used for quantitative real‐time PCR. Five sets of primers were designed, including two amplicons without KLF5 binding sites and three amplicons with KLF5 binding sites. The details of the primers were provided in Table S1.

### 2.16 Co‐immunoprecipitation assay

MIA‐PaCa‐2 and PANC‐1 cells were lysed in ice‐cold co‐immunoprecipitation (Co‐IP) lysis buffer (Beyotime, Wuhan, China) and were then incubated on ice for 10 minutes. The insoluble material was pelleted at 13 000 × g for 10 minutes at 4°C. The supernatant was pre‐cleaned by protein A/G PLUS‐Agarose (Santa Cruz Biotechnology, Santa Cruz, CA, USA), and the aliquots were immunoprecipitated with an antibody against KLF5 (21017‐1‐AP; Thermo Fisher Scientific), followed by incubation with protein A/G PLUS‐Agarose beads overnight at 4°C. The immunoprecipitated complexes were washed, and the precipitated proteins were then analysed by Western blot analysis as described above. The input was used as a positive control.

### 2.17 Statistical analysis

Statistical analysis was performed using GraphPad Prism 8.1.2 (GraphPad Inc, La Jolla, CA, USA) and SPSS 25.0 software package (SPSS Inc, Chicago, IL, USA). For multiple group comparison, one‐way ANOVA with post hoc Tukey's multiple comparisons test was performed. For two‐group comparison, Welch's unequal variances *t* test was applied to detect the differences. Kaplan‐Meier survival curves were generated using GraphPad Prism. Patients were separated into two groups by median gene expression. Log‐rank test was performed to determine the significance of the difference between the survival curves. Correlation analysis was performed by calculating Pearson's coefficient. *P* < 0.05 was considered to be statistically significant.

## 3 RESULT

### 3.1 Aberrant *PLA2G16* expression was associated with unfavourable survival of PAAD

*PLA2G16* has two protein‐coding transcripts encoding the same protein. By comparing the transcriptional profiles of *PL2G16* between TCGA‐PAAD (N = 178) and GTEx‐pancreas (N = 167), we confirmed that total *PL2G16* expression and the two protein‐coding transcripts ENST00000415826.1 and ENST00000323646.9 were significantly up‐regulated in the tumour tissue (Figure 1A,B). Then, we checked the association between *PLA2G16* expression and the clinicopathological parameters of PAAD patients. Results showed that *PLA2G16* expression was significantly increased in the higher histological grade of tumours (G2 and G3/G4) compared to the low‐grade tumours (G1) (Figure 1C). Besides, its expression might also be related to the history of chronic pancreatitis (Figure 1D). Then, we examined PLA2G16 protein expression using IHC staining data from the HPA. In normal pancreas, PL2G16 expression was usually low in exocrine glandular cells (Figure 1E, left) and was negative in duct cells (Figure 1E, left, enlarged areas). In comparison, among 10 cases of PAAD in the HPA, 4 cases had high or medium expression (Figure 1E, right and G). To validate PLA2G16 expression, we conducted IHC using commercially available human tissue array. Results showed that PLA2G16 expression was low in PAAD adjacent normal tissues, but was increased in pancreatitis and PAAD tissues (Figure 1F). 9/16 (56.3%) PAAD cases had high or medium PLA2G16 expression (Figure 1H). Then, we checked whether *PLA2G16* expression was associated with the prognosis of PAAD. K‐M survival analysis indicated that patients with high *PLA2G16* (top 50%) expression had significantly worse PFS, DSS and OS compared to the patients with low *PLA2G16* (bottom 50%) expression (Figure 1I‐K).

### 3.2 PLA2G16 enhances pancreatic cell growth in vitro and in vivo

**FIGURE 2:**
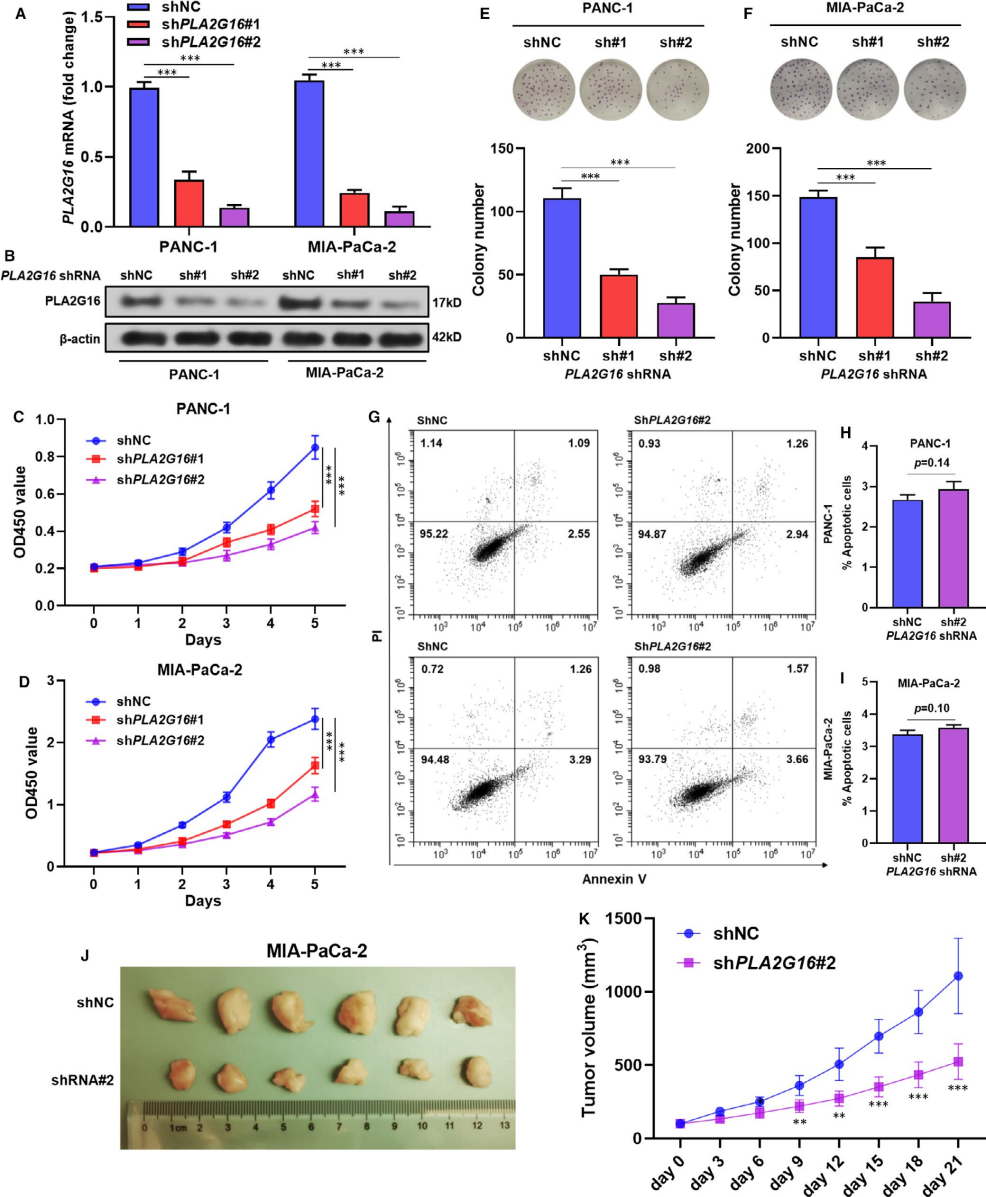
PLA2G16 enhances pancreatic cell growth in vitro and in vivo. A and B, quantitative real‐time PCR (qRT‐PCR) (A) and Western blot (B) analysis of *PLA2G16* expression in PANC‐1 and MIA‐PaCa‐2 cells 48 h (qRT‐PCR)/72 h (Western blot) after lentiviral‐mediated *PLA2G16* knockdown. C and D, cell count kit assay of the proliferation of PANC‐1 (C) and MIA‐PaCa‐2 (D) cells with or without *PLA2G16* knockdown. E and F, Representative image (up) and quantification (down) of colony formation of PANC‐1 (E) and MIA‐PaCa‐2 (F) cells with or without *PLA2G16* knockdown. G‐I, Representative image (G) and quantification (H, I) of flow cytometric analysis of apoptotic PANC‐1 and MIA‐PaCa‐2 cells 48 h after lentiviral‐mediated *PLA2G16* knockdown. J and K, Representative images (J) of xenograft tumour developed by MIA‐PaCa‐2 cells with or without *PLA2G16* knockdown and the corresponding tumour growth curve (K)

To explore the potential influence of PLA2G16 on the growth of PAAD cells, PANC‐1 and MIA‐PaCa‐2 cells were subjected to lentivirus‐mediated *PLA2G16* knockdown (Figure S2A; Figure 2A,B). CCK‐8 assay showed that *PLA2G16* knockdown significantly decreased the viability of PANC‐1 and MIA‐PaCa‐2 cells (Figure 2C,D). Colony formation was also reduced by PLA2G16 knockdown (Figure 2E,F). Flow cytometric analysis using Annexin V/PI showed that *PLA2G16* knockdown did not influence apoptosis (Figure 2G‐I). These findings suggested that the growth inhibition effect might be a result of proliferation inhibition, but not increased apoptosis. To further illuminate the role of PLA2G16 in in vivo tumour growth, MIA‐PaCa‐2 cells with or without *PLA2G16* inhibition were used to generate xenograft tumour model. All mice developed xenograft tumours (Figure 2J). *PLA2G16* inhibition remarkably impaired tumour growth (Figure 2K).

### 3.3 PLA2G16 enhances glycolysis in pancreatic cancer cells

**FIGURE 3:**
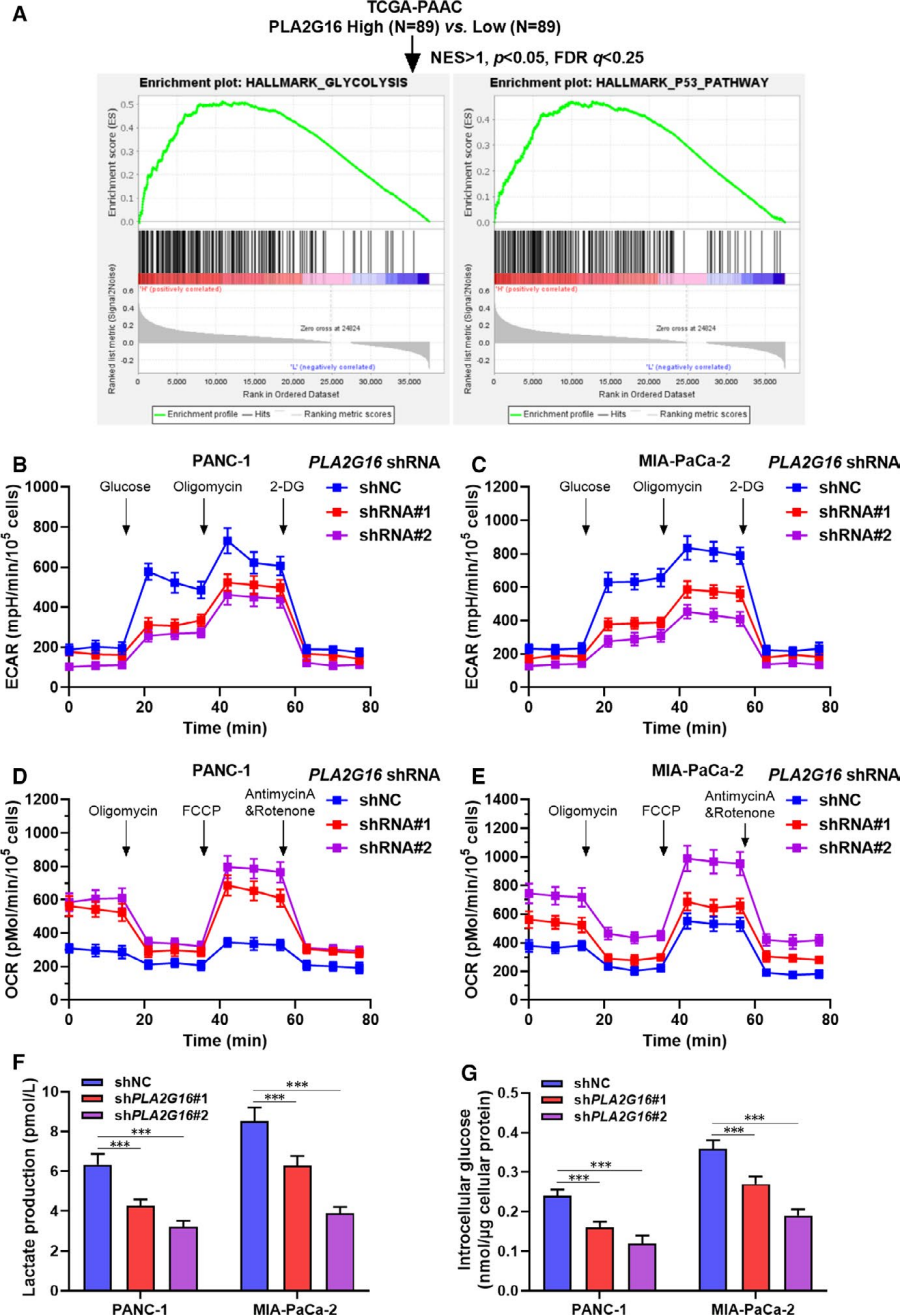
PLA2G16 enhances glycolysis in pancreatic cancer cells. A, Group stratification for single‐gene gene set enrichment analysis in primary pancreatic adenocarcinoma from The Cancer Genome Atlas (TCGA) Pan‐Cancer and summary of gene set enrichment in the high *PLA2G16* expression group. B‐E, Measurement of extracellular acidification rates (ECAR) (B, C) and oxygen consumption rate (OCR) (D, E) in PANC‐1 (B, D) and MIA‐PaCa‐2 (C, E) cells with or without *PLA2G16* knockdown. F and G, Measurement of lactate production (F) and glucose uptake (G) in PANC‐1 and MIA‐PaCa‐2 cells with or without *PLA2G16* knockdown. Intracellular glucose levels were measured and normalized based on protein concentration. FCCP, carbonyl cyanide 4‐(trifluoromethoxy) phenylhydrazone

Gene set enrichment analysis was performed to explore the diversity of gene set enrichment between tumours with high and low *PLAG16* expression. Results showed that the high expression group had elevated genes enriched in glycolysis and p53 pathways (Figure 3A). Then, the change in aerobic glycolysis was measured using Seahorse XF analyzer. Results showed that the ECAR significantly decreased in both PANC‐1 and MIA‐PaCa‐2 cells with *PLA2G16* knockdown, indicating inhibited glycolytic process (Figure 3B,C). Aerobic glycolysis is associated with decreased OCR in cells. In this study, we observed that *PLA2G16* inhibition resulted in decreased OCR in both PANC‐1 and MIA‐PaCa‐2 cells (Figure 3D,E), suggesting that PLA2G16 negatively regulated mitochondrial respiration. Besides, *PLA2G16* inhibition also led to a substantial decrease in lactate production and glucose uptake in PANC‐1 and MIA‐PaCa‐2 cells (Figure 3F,G). Collectively, these findings suggest that PLA2G16 acted as a positive regulator of aerobic glycolysis in PAAD cells.

### 3.4 Both mutant p53 and KLF5 increase *PLA2G16* expression in pancreatic cancer

**FIGURE 4:**
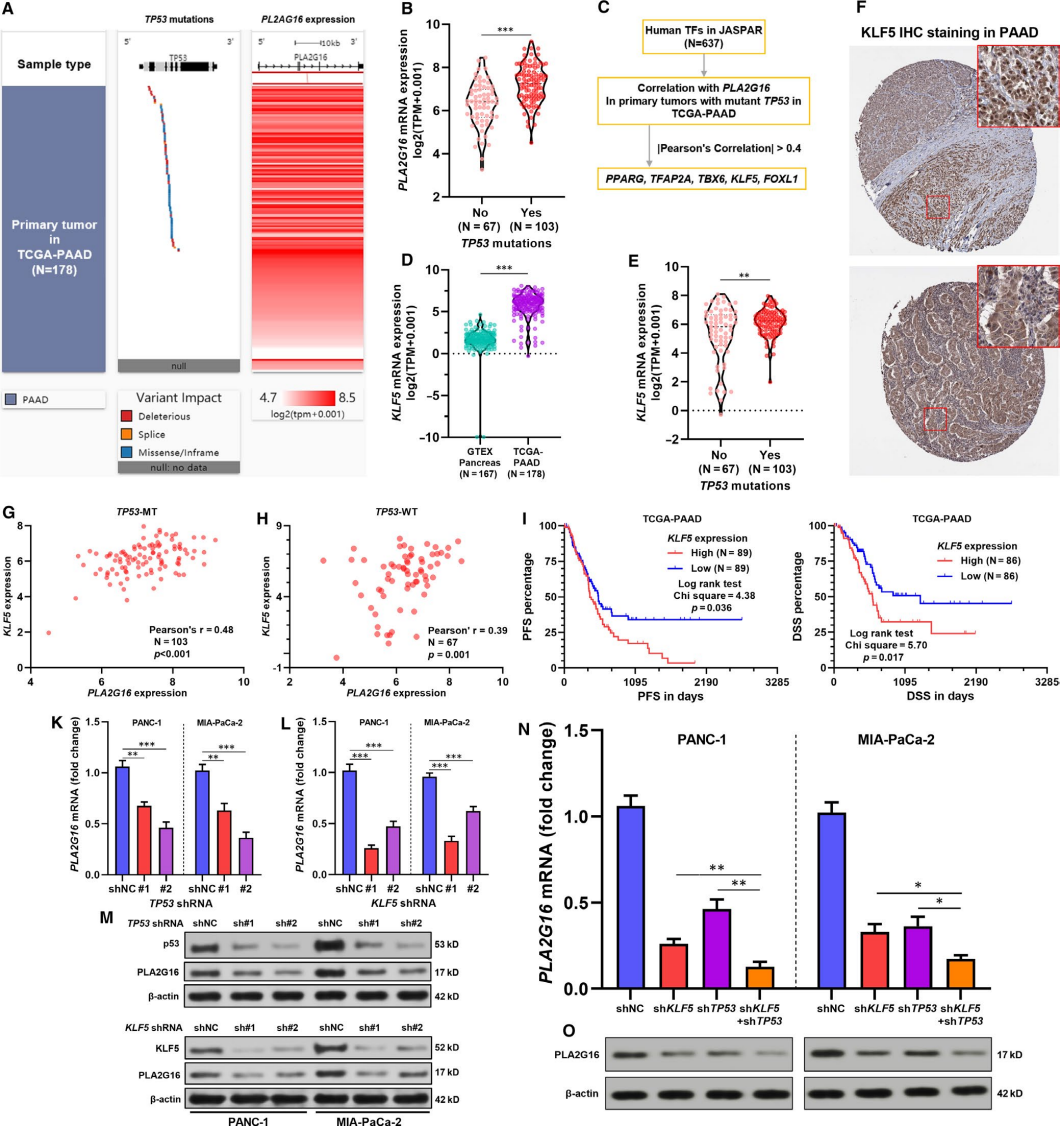
Both mutant p53 and KLF5 increase *PLA2G16* expression in pancreatic cancer. A, A heatmap showing *TP53* mutation and *PLA2G16* expression in pancreatic adenocarcinoma (PAAD) cases in The Cancer Genome Atlas (TCGA). B, A violin plot chart comparing *PLA2G16*expression between PAAD cases with or without *TP53* mutations. C, Flow chart showing the screening process to identify *PLA2G16* correlated TFs in *TP53* mutant PAAD cases. D and E, Comparison of *KLF5* mRNA expression between normal pancreas in GTEx and PAAD in TCGA (D) and between PAAD cases with or without *TP53* mutations (E). F, Representative images of KLF5 protein expression in PAAD tissue. Image credit: Human Protein Atlas, from: https://www.proteinatlas.org/ENSG00000102554‐KLF5/pathology/pancreatic+cancer. G and H, Plot charts showing the correlation between *PLA2G16* and *KLF5* mRNA expression in *TP53* mutant (G) and *TP53* wild‐type (H) PAAD cases. I and J, K‐M survival analysis of PFS (I) and DSS (J) in PAAD cases in TCGA Pan‐Cancer, by median *KLF5* expression separation. K and L, Quantitative real‐time PCR (qRT‐PCR) analysis of *PLA2G16* mRNA expression in PANC‐1 and MIA‐PaCa‐2 cells 48 h after lentiviral‐mediated *TP53* (K) or *KLF5* (L) inhibition. M, Western blot analysis of p53, KLF5 and PLA2G16 protein expression 72 h after lentiviral‐mediated *TP53* (up) or *KLF5* (down) inhibition. N and O, qRT‐PCR (M) and Western blot assay (O) of *PLA2G16* expression in PANC‐1 and MIA‐PaCa‐2 cells with *TP53* and *KLF5* inhibition (sh*TP53*#2 and shKLF5#1) separately or in combination

Since mutant p53 indirectly drives *PLA2G16* up‐regulation in osteosarcoma,^16^ we checked their association in PAAD using data from TCGA. 170/178 cases in TCGA‐PAAD had *TP53* mutation data. *TP53* mutation was quite common (103/170, 60.6%) (Figure 4A). *TP53* mutation group had significantly higher *PLA2G16* expression compared to the group without *TP53* mutation (Figure 4B). To explore other potential transcriptional factors involved in *PLA2G16* dysregulation in PAAD, we checked the correlations of human TFs in JASPAR database (N = 637) with *PLA2G16* expression in tumours harbouring *TP53* mutation (Figure 4C). By setting |Pearson's *r*| > 0.4 as the cut‐off, we identified 5 TF genes correlated with *PLA2G16* expression, including *PPARG, TFAP2A, TBX6, KLF5* and *FOXL1* (Figure 4C). Among them, *KLF5* is an oncogene in pancreatic cancer^24^, ^25^, ^26^ and interacts with p53 in acute lymphoblastic leukaemia.^27^ Using RNA‐seq data in GTEx and TCGA, we confirmed that KLF5 was significantly up‐regulated in PAAD tissues (Figure 4D). *TP53* mutant tumours also had significantly higher *KLF5* expression (Figure 4E). IHC staining data in HPA confirmed that *KLF5* was expressed at the protein level in PAAD tissues (Figure 4F). Interestingly, by examining the correlation between *KLF5* and *PLA2G16* expression in *TP53* wild‐type and mutant tumours respectively, we observed a higher level of correlation in *TP53* mutant tumours (Pearson's *r*, 0.48 vs 0.39) (Figure 4G,H). K‐M survival analysis indicated that PAAD patients with high *KLF5* expression also had significantly worse PFS and DSS (Figure 4I,J).

To further explore the potential influence of mutant p53 and KLF5 on *PLA2G16* expression, PANC‐1 and MIA‐PaCa‐2 cells were subjected to *TP53* or *KLF5* inhibition respectively (Figure S2B,C). *TP53* or *KLF5* inhibition significantly reduced *PLA2G16* expression at both mRNA and protein levels (Figure 4K‐M). Simultaneous *TP53* and *KLF5* inhibition had a synergistic effect on lowering *PLA2G16* expression (Figure 4N,O).

### 3.5 KLF5 binds to the *PLA2G16* promoter and activates its transcription

**FIGURE 5:**
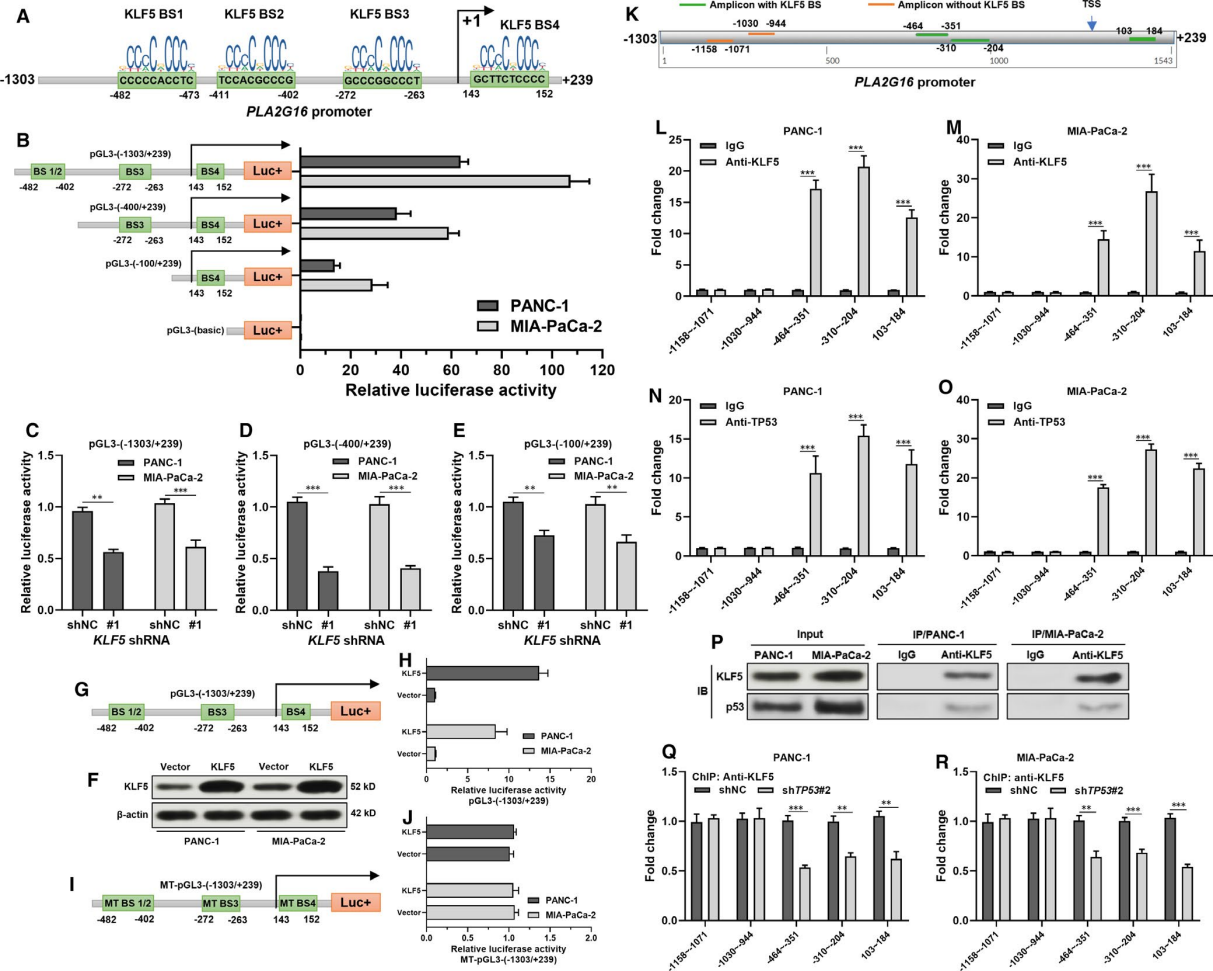
Mutant p53 enhances KLF5‐induced *PLA2G16* transcriptional activation. A, Predicted binding sites of KLF5 in the promoter region of *PLA2G16*. B, The promoter activity of the *PLA2G16* gene was measured using a dual‐luciferase reporter assay. PANC‐1 and MIA‐PaCa‐2 cells were transfected with pGL3‐basic or reporter constructs carrying different lengths of the 5ʹ‐flanking region of the *PLA2G16* promoter as indicated. C‐E, *KLF5* depletion reduced the activity of the *PLA2G16* promoter. PANC‐1 and MIA‐PaCa‐2 cells with or without lentiviral‐mediated *KLF5* inhibition were transfected reporter constructs carrying pGL3‐(−1303/+239) (C), pGL3‐(−400/+239) (D) and pGL3‐(−100/+239) (E). 24 h later, luciferase activity was determined. F, Western blot assay of KLF5 expression 48 h after lentiviral‐mediated overexpression. G‐J, 24 h after infection with lenti‐KLF5 or vector, PANC‐1 and MIA‐PaCa‐2 cells were transfected reporter constructs carrying pGL3‐(−1303/+239) (G‐H) or mutant‐pGL3‐(−1303/+239) with mutant sequences (C to A) of the four binding sites (I, J). 24 h later, luciferase activity was determined. K, Schematic image showing the location of the designed primer sets for ChIP‐quantitative polymerase chain reaction (qPCR) assay, by anti‐KLF5 immunoprecipitation. L‐O, ChIP‐qPCR assays were performed using anti‐KLF5 (L, M) or anti‐TP53 (N, O) and control IgG antibodies in PANC‐1 (L, N) and MIA‐PaCa‐2 (M, O) cells. Fold enrichment of the indicated PLA2G16 promoter segments was calculated. P, Co‐IP assay to explore the potential binding between KLF5 and p53 in MIA‐PaCa‐2 and PANC‐1 cells. Q and R, ChIP‐qPCR assays were performed using anti‐KLF5 in MIA‐PaCa‐2 (Q) and PANC‐1 (R) cells with or without *TP53* inhibition. Fold enrichment of the indicated regions of the *PLA2G16* promoter was calculated. *P < .05; **P < .01; ***P < .001

Via scanning the promoter sequence of *PLA2G16* promoter, we observed four high potential KLF5 binding sites (Figure 5A). To explore the potential influence of KLF5 on the promoter activities of *PLA2G16*, different lengths of the 5ʹ flanking region of *PLA2G16,* including –1303/+239, −400/+239, and −100/+239, were cloned into the pGL3‐basic plasmid and transiently transfected into PANC‐1 and MIA‐PaCa‐2 cells. Dual‐luciferase reporter assay showed that luciferase constructs with KLF5 binding sites had significantly higher luciferase activity compared to the pGL3‐basic plasmid (Figure 5B). pGL3‐(1303/+239) with integrate *PLA2G16* promoter had the highest luciferase activity, while the constructed with truncated KLF5 binding sites had significantly decreased luciferase activity (Figure 5B). Then, PANC‐1 and MIA‐PaCa‐2 cells with or without lentivirus‐mediated *KLF5* inhibition were transfected with different recombinant pGL3 constructs with truncated *PLA2G16* promoter fragments. The group with *KLF5* inhibition had significantly lower luciferase activity (Figure 5C‐E). To further validate the promoter activating effects of KLF5, PANC‐1 and MIA‐PaCa‐2 cells were subjected to lentivirus‐mediated *KLF5* overexpression (Figure 5F). A reporter plasmid carrying mutant sequences of the four binding sites was also generated (MT‐pGL3‐(1303/+239); Figure 5I). Then, PANC‐1 and MIA‐PaCa‐2 cells with or without *KLF5* overexpression were transfected with pGL3‐(1303/+239) (Figure 5G) or MT‐pGL3‐(1303/+239) (Figure 5I). Dual‐luciferase assay confirmed that KLF5 overexpression only activated the wild type but not the mutant promoter sequence (Figure 5G‐J).

Then, five pairs of primers targeting the *PLA2G16* promoter were designed for ChIP‐qPCR assay (Figure 5K). The three amplicons covering KLF5 binding sites were significantly enriched upon anti‐KLF5 immunoprecipitation in both MIA‐PaCa‐2 and PANC‐1 cells (Figure 5L,M). But this phenomenon was not observed in the two amplicons without KLF5 binding sites (Figure 5L,M). Similar results were seen using anti‐TP53 immunoprecipitation (Figure 5N,O).

KLF5 has physical interaction with p53 in acute lymphoblastic leukaemia.^27^ Co‐IP assay in the current study confirmed the interactions between KLF5 and mutant p53 in MIA‐PaCa‐2 and PANC‐1 cells (Figure 5P). Following ChIP‐qPCR assay indicated that inhibiting endogenous mutant p53 reduced the enrichment of the KLF5‐binding PLA2G15 promoter segments (Figure 5Q,R). These findings suggested that mutant p53 directly interacted with KLF5 and enhanced the binding of KLF5 to the *PLA2G16* promoter.

### 3.6 *PLA2G16* up‐regulation was also associated with gene‐level copy amplification and hypomethylation of certain 5’‐cytosine‐phosphodiester bond‐guanine‐3’ (CpG) sites

**FIGURE 6:**
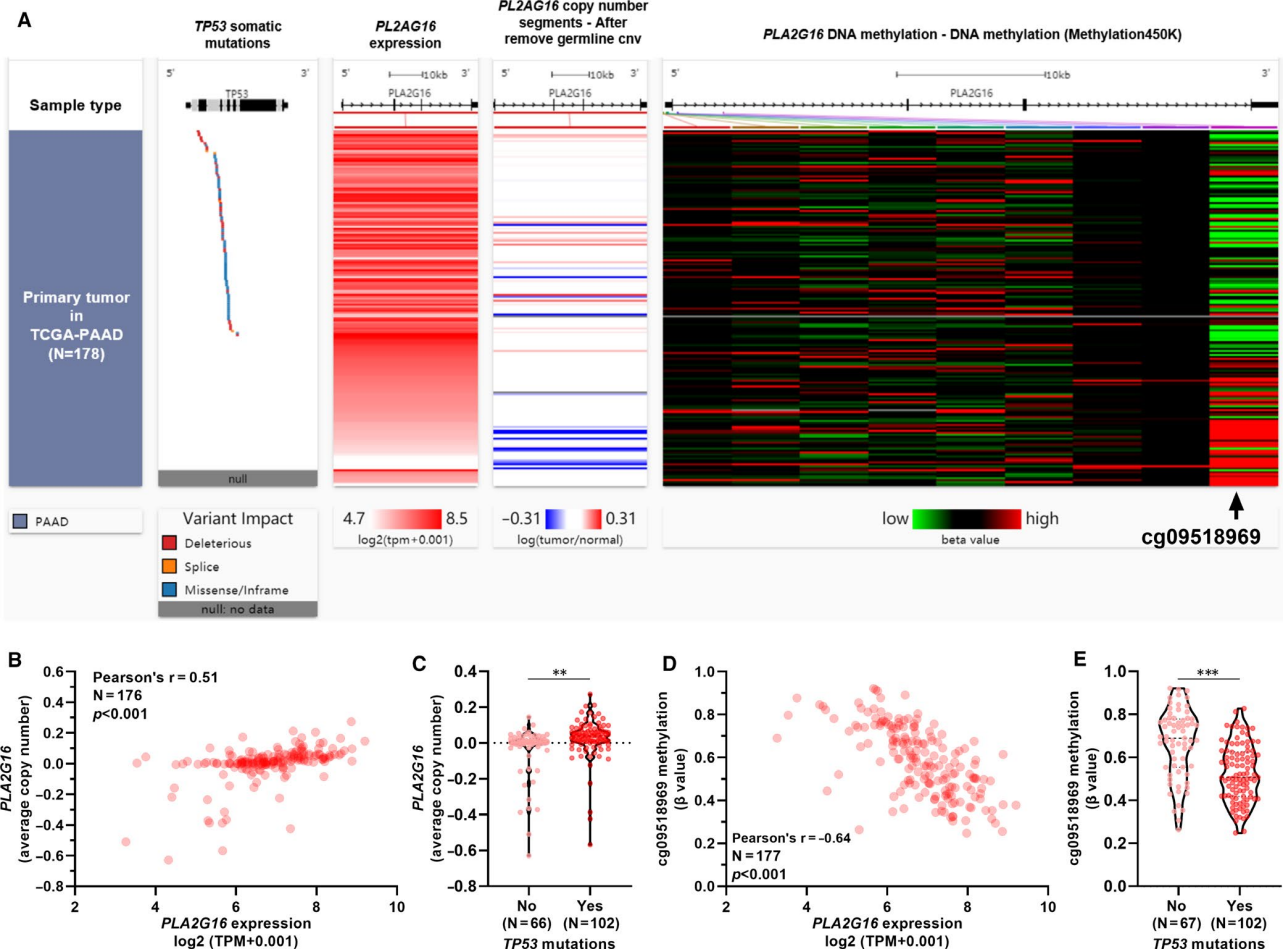
*PLA2G16* up‐regulation was also associated with gene‐level copy amplification and hypomethylation. A, A heatmap showing *PLA2G16* expression, copy number alteration and methylation level of nine 5’‐cytosine‐phosphodiester bond‐guanine‐3’ sites in pancreatic adenocarcinoma (PAAD) cases in The Cancer Genome Atlas (TCGA). B, A plot chart showing the correlation between *PLA2G16* expression and its copy number in 176 PAAD cases in TCGA. C, Comparison of *PLA2G16* expression copy number between *TP53* wild‐type and mutant PAAD cases. D, A plot chart showing the correlation between *PLA2G16* expression and the β‐value of cg09518969 in 177 PAAD cases in TCGA. E, Comparison of cg09518969 methylation value between *TP53* wild‐type and mutant PAAD cases

To explore whether other genetic and epigenetic mechanisms are involved in *PLA2G16* dysregulation, we check copy number alteration and methylation profile of CpG sites within its gene locus. Correlation analysis showed that *PLA2G16* expression was moderately and positively correlated with its copy number (Pearson's *r* = 0.51, *P* < 0.001) (Figure 6A‐B). Among 9 CpG sites within its gene locus, cg09518969 methylation was strongly and negatively correlated with *PLA2G16* expression (Pearson's *r* = −0.64, *P* < 0.001; Figure 6A,D). We then checked the *PLA2G16* copy number and cg09518969 methylation in *TP53* wild‐type and mutant cases, respectively. Results showed that the mutant *TP53* group had significantly higher *PLA2G16* copy number and lower cg09518969 methylation (Figure 6C,E).

## 4 DISCUSSION

In this study, we found that *PLA2G16* expression was significantly up‐regulated in PAAD and was associated with unfavourable prognosis. As a phospholipase, PLA2G16 up‐regulation increases the production of LPAs and FFAs. The proliferation enhancing effects of lipids on PAAD cell growth was observed on several levels. Firstly, normal pancreatic cells have better capability to utilize lipids as an energy source compared to other cell types such as liver cells.^28^ The fatty acid exposure increases medium‐chain acyl‐coenzyme A dehydrogenase expression and cell proliferation of MIA‐PaCa‐2 cells,^29^ suggesting that fatty acids and lipids provide an energy source for PAAD cells. Secondly, lipid‐derived signal transductors are important signal mediators, which are involved in multiple growth‐regulatory pathways. PAAD cells secrete several types of cytokines and growth factors such as interleukin (IL)‐6, IL‐8, basic fibroblast growth factor, nerve growth factor and vascular endothelial growth factor. They are implicated in cell proliferation via paracrine or autocrine mechanisms.^30^, ^31^, ^32^ Therefore, endogenous LPAs and FFAs help transduct the signallings and accelerate cellular growth. Our in vitro and in vivo studies confirmed that PLA2G16 acted as a tumour growth‐promoting factor in PAAD.

Gene set enrichment analysis analysis in the current study indicated that PAAD cases with high *PLA2G16* expression had significantly up‐regulated genes enriched in glycolysis and p53 pathways. Following functional assay showed that its expression enhances aerobic glycolysis in PAAD cells. Like all the other cancers, PAAD cells have metabolic reprogramming, including enhanced glycolysis, diverted glutamine consumption, anomalous pentose phosphate pathway and autophagy.^33^ The metabolic addiction of tumour cells supports excessive energy consumption required for rapid cell proliferation. Recent studies indicated that the addiction of glycolysis is a result of a multitude of oncogenic aberrations, which is associated with enhanced glucose uptake and metabolic rate.^33^, ^34^ Although glycolysis is much less efficient in energy production than mitochondrial respiration, it provides several advantages for the tumorigenic properties. Firstly, aerobic glycolysis enables PAAD cells to thrive independently of an inconstant oxygen diffusion.^33^ Secondly, lactate efflux helps generate an acid environment that supports the invasion of PAAD cells and is also a glucose‐alternative carbon source of neighbouring oxygenated cancer cells.^35^ Thirdly, increased lactate concentration contributes to an enhanced immunosuppressive tumour microenvironment, at least by inhibiting the activation of cytotoxic tumour‐infiltrating T lymphocytes and NK cells and supporting the generation of myeloid‐derived suppressor cells.^36^

Till now, the dysregulation of *PLA2G16* in tumours is still mysterious. One previous study found that mutant p53 enhances *PLA2G16* expression through binding to E26 transformation‐specific motifs in the *PLA2G16* promoter indirectly via ETS2.^16^ In this study, we revealed that mutant p53 also promoted *PLA2G16* expression in PAAD. Mutant p53 directly bound with KLF5, which transcriptionally activated *PLA2G16* via promoter binding. Inhibition of mutant p53 impaired the transcriptional activating effects of KLF5. Based on these observations, we infer that mutant p53 acts as a co‐factor of KLF5 in binding to the *PLA2G16* promoter and activating its transcription. The oncogenic effects of mutant p53 and KLF5 in PAAD have been widely reported by previous studies. Mutant p53 exerts gain‐of‐function activities independent of their effects on wild‐type p53. It maintains the premetastatic phenotype of PAAD by inducing the expression of platelet‐derived growth factor receptor b.^37^, ^38^ It also activates a positive feedback loop between NRF2 and p62 to induce chemoresistance.^39^ Besides, mutant p53 prevents glyceraldehyde‐3‐phosphate dehydrogenase nuclear translocation, thereby generating a favourable cellular environment for glycolysis.^40^ KLF5 is a key TF linking cellular senescence regulation in PAAD^41^ and is among a 25 gene panel significantly associated with PAAD risk.^42^ Its expression is induced by IL‐1 beta or hypoxic microenvironment in PAAD and activates the expression of GLUT‐1, Survivin and platelet‐derived growth factor‐A.^25^ Besides, KLF5 promotes the expression of E2F1, cyclin D1 and Rad51, while inhibits the expression of p16, thereby facilitating G1/S progression of PAAD cells.^24^ Inhibiting KLF5 increases the expression of *NDRG2*, reduces the activation of STAT3, retards acinar‐to‐ductal metaplasia and impairs the formation of pancreatic intraepithelial neoplasia in mice model.^26^ Findings in the current study expanded our understanding of the downstream regulation of mutant p53 and KLF5 in PAAD.

*PLA2G16* was found as a methylation responsive gene in prostate cancer.^43^ Using copy number and methylation data in TCGA, we also observed that *PLA2G16* expression was positively correlated with its copy number, but was negatively correlated with the methylation of a CpG site. The mutant *TP53* group had significantly higher *PLA2G16* copy number and lower cg09518969 methylation compared to the TP53 wild‐type group. Previous studies reported that TP53 mutations lead to genomic instability by facilitating the selection of some hypomethylated and gene copy‐amplified cells.^44^, ^45^ Collectively, these findings suggest that *PL2G216* dysregulation is a result of accumulated genetic and epigenetic alterations.

## 5 CONCLUSION

This study identified a novel regulative effect of *PLAG16* on tumour growth and aerobic glycolysis of PAAD cells. Besides, we demonstrated that KLF5 directly binds to the *PLA2G16* promoter and activates its expression, while mutant p53 enhances the effects of KLF5 via interacting with it. The newly established mutant p53/KLF5‐PLA2G16 regulatory axis in PAAD provides a strong rationale for exploring new therapeutic targets in patients with mutant p53.

## CONFLICTS OF INTEREST

The authors have no conflict of interest.

## AUTHOR CONTRIBUTION

**Wei Xia:** Conceptualization (equal); Formal analysis (equal); Methodology (equal); Software (equal); Validation (equal); Writing‐original draft (equal); Writing‐review & editing (equal). **Hansong Bai:** Formal analysis (equal); Investigation (equal); Software (equal); Validation (equal); Visualization (equal); Writing‐original draft (equal); Writing‐review & editing (equal). **Ying Deng:** Data curation (equal); Methodology (equal); Software (equal); Writing‐original draft (equal); Writing‐review & editing (equal). **Yi Yang:** Conceptualization (equal); Formal analysis (equal); Methodology (equal); Project administration (equal); Supervision (equal); Validation (equal); Visualization (equal); Writing‐original draft (equal); Writing‐review & editing (equal).

## Supporting information

Fig S1

Fig S2

Table S1

Figure Legends

## Data Availability

All data generated or analysed during this study are included in this published article and in Supporting Information files.
