# Supplementary material for: PLA2G16 is a mutant p53/KLF5 transcriptional target and promotes glycolysis of pancreatic cancer
Source: J Cell Mol Med. 2020 Sep 27;24(21):12642–55. doi: 10.1111/jcmm.15832 (PMC7686977; doi:10.1111/jcmm.15832)
Supplement: Supplementary file 1 — Fig S1 [file JCMM-24-12642-s001.docx]

>HPRM34796 NM_001128203;name=PLA2G16;Entrez_ID=11145;Genome=hg38;chr11-:63615772-63614230;TSS=63614469;Upstream=1303,Downstream=239;Length=1543;

TCGCTTGCCCGGAAGGCAGAGGTTGCAGTGAGCTGAGATAGAGCCACTGCACTCCAGCCT

GGGTGACAGAGTGAGACTGTCTCAAAAAAAAAAAAACAAAAAACCCCAAAAAGTAAAAAA

TGAAAAAAGTTATTTCAACAAGGTCTGTACAGAATTCTTTCCGTCTCAACATCTCCTCTT

TGAGGATAAGGATGTTGCTTTTTCTTCTAGTATAAGGAAGGTGTCCTTCAACATGGAAAT

TTCATCTGCTTTTAAGACACAGAAAAGGGGTCTTCTTGCACCTGCTGAGTTTTAAATGCC

TTTAATTCAAAACAGCCAATATGCCATGGTGGTGTGTATATACACACACACACACACACA

CACACATATACACACATATATACACATATATATACACACATATATATGCACACATATATA

TACACACATATATACACACATATATACACACATATATACACACATATATACACACATATA

TATACACATATATACACATATATACACACATATATATACACACACATATATACACATATA

TACACATATATACACATATATATACACACATATATACACACATATATATACACACATATA

CACACATATATACACATATATACACACATATACACATATACATATATACACACATATATA

CACACATATATACACATATATACACATATATACACACATATATACACACATATATATACA

CATATATATACATATATATACACACATATATATATTTTGAGACTGAGTTTCGCTTTGTTG

CACAGGCTAGAGTGCAGTGGCGCGATCTTGGCTCACTGCAA**CCCCCACCTC**CCGGGCTCA

AGTGATTCTCCTGCCTCAGCCTCCCGAGTAGCTGGGACTACAGGCGCATGCC**TCCACGCC**

**CG**GCTAATTTTTTGCATTTTTAGTAGAGACGGGGTTTCATCGTGTTAGCCAGCATGGTCT

CGATCTCTTGACCTCGTGATCTGCCCGCCTCGGCCTCCCAAAGTGCTGAGATTACAGGCG

TGAGCCACCGC**GCCCGGCCCT**TGGTGGTATATTTTTAACTCCTTCAGTTTTTAAACTATA

AGCCCATTCTTGAGTGAAGGCGAAAGTAAACCCATCATGGCCCTGCAGTGTGATGTGTGT

GCAGAGGTCGAGTGTGTGCGACTCCTGGATGCTGGGCGCGCAGGGCATGGGTGAGGCGGG

AAGAGGCGGTGCCGGGGGCGCGGGCGTCCTGCAGTCGCCGGGCTCGGGACCGGGGCCGGG

CGCTCTGCGAGGCTCTCATTAGCCGGCGGCGCGGGGAGGGGCCGGGTGACCTCACGCCGG

CCCGGCCACCGCGGCCATTAGACCCGGTCCAATTGCTGGGGCTGCAGCGCTGCCTCCGAG

ACCGCGAGGTGGGTGGATCGGGTCTTCCTGGAAGGGTGCGATAAGGCCGGGCGAGGTGCC

TGGGAT**GCTTCTCCCC**TTCCGCGAGGAAGAGATCTAATTGGGTAGGGCGGGTGTAGACTA

GCCTGCCGAGCCGCCCGCTGGCACCTGCAGCCTCCTGGGCGCC
