# Supplementary figures and images for: PLA2G16 is a mutant p53/KLF5 transcriptional target and promotes glycolysis of pancreatic cancer
Source: J Cell Mol Med. 2020 Sep 27;24(21):12642–55. doi: 10.1111/jcmm.15832 (PMC7686977; doi:10.1111/jcmm.15832)

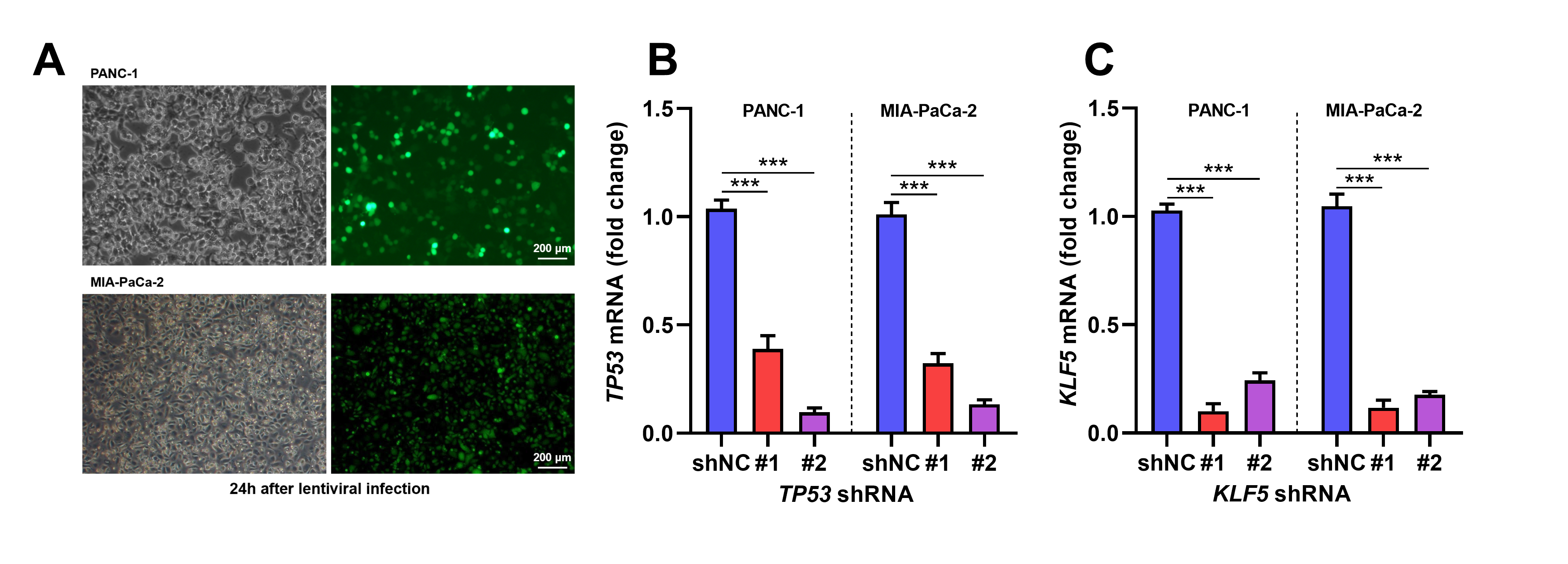

Supplement: Supplementary file 2 — Fig S2 [file JCMM-24-12642-s002.tif]
