# Supplementary material for: PLA2G16 is a mutant p53/KLF5 transcriptional target and promotes glycolysis of pancreatic cancer
Source: J Cell Mol Med. 2020 Sep 27;24(21):12642–55. doi: 10.1111/jcmm.15832 (PMC7686977; doi:10.1111/jcmm.15832)
Supplement: Supplementary file 3 — Table S1 [file JCMM-24-12642-s003.docx]

**Supplementary Table 1. Primer sets for ChIP-qPCR assay**

| **Assay set with**  **KLF5 binding** | **Type** | **Sequence 5’-3’** | **Amplicon length** | **Amplicon position** |
| --- | --- | --- | --- | --- |
|  | Forward Primer | CCTCCCAAAGTGCTGAGATTAC | 107 | -310 |
|  | Reverse Primer | CCTTCACTCAAGAATGGGCTTAT |  | -204 |
|  |  |  |  |  |
|  | Forward Primer | CCTGGAAGGGTGCGATAAG | 82 | 103 |
|  | Reverse Primer | GCCCTACCCAATTAGATCTCTTC |  | 184 |
|  |  |  |  |  |
|  | Forward Primer | AAGTGATTCTCCTGCCTCAG | 114 | -464 |
|  | Reverse Primer | GCTGGCTAACACGATGAAAC |  | -351 |
| **Assay set without KLF5 binding** | **Type** | **Sequence 5’-3’** | **Amplicon length** | **Amplicon position** |
|  | Forward Primer | TCTTGCACCTGCTGAGTTT | 87 | -1030 |
|  | Reverse Primer | TGTGTGTGTGTGTGTGTGTA |  | -944 |
|  |  |  |  |  |
|  | Forward Primer | TGTACAGAATTCTTTCCGTCTCA | 88 | -1158 |
|  | Reverse Primer | TGTTGAAGGACACCTTCCTTAT |  | -1071 |
