# Supplementary material for: PLA2G16 is a mutant p53/KLF5 transcriptional target and promotes glycolysis of pancreatic cancer
Source: J Cell Mol Med. 2020 Sep 27;24(21):12642–55. doi: 10.1111/jcmm.15832 (PMC7686977; doi:10.1111/jcmm.15832)
Supplement: Supplementary file 4 — Figure Legends [file JCMM-24-12642-s004.docx]

**Supplementary Figure 1. The promoter sequence of *PLA2G16***

**Supplementary Figure 2. The promoter sequence of *PLA2G16***. **A.** Representative bright-field (left) and green fluorescent (right) images of PANC-1 and MIA-PaCa-2 cells 24h after infection with lentiviral sh*PLA2G16*. **B-C.** QRT-PCR analysis of *TP53* mRNA (B) and *KLF5* mRNA (C) expression in PANC-1 and MIA-PaCa-2 cells 48h after lentiviral-mediated *TP53* or *KLF5* inhibition.
